# Colorado Immersion Training in Community Engagement: Ten years of learning and doing

**DOI:** 10.1017/cts.2024.658

**Published:** 2024-12-05

**Authors:** Leslie A. Wright, Reginaldo Garcia, Mary E. Fisher, Kaylee Gordon, Donald E. Nease, Elizabeth Sweitzer, Crystal LoudHawk-Hedgepeth, Charlene Barrientos Ortiz, Montelle Taméz, Lorenzo Ramírez, Linda Zittleman, Bruno Walther Santos Sobral

**Affiliations:** 1 Kaiser Permanente Colorado Institute for Health Research, Aurora, CO, USA; 2 University of Colorado Clinical and Translational Sciences Institute – Community Engagement and Health Equity Core, Aurora, CO, USA; 3 Department of Family Medicine, University of Colorado School of Medicine, Aurora, CO, USA; 4 The Evaluation Center, University of Colorado Denver, Denver, CO, USA; 5 Department of Community and Behavioral Health, Colorado School of Public Health, Aurora, CO, USA; 6 Department of Microbiology, Immunology and Pathology, Colorado State University, Fort Collins, CO, USA; 7 Department of Biostatistics and Informatics, Colorado School of Public Health, Aurora, CO, USA

**Keywords:** Community-Based Participatory Research, community–academic partnerships, Clinical and Translation Science, participatory action research, Community Research Liaisons

## Abstract

The Colorado Immersion Training in Community Engagement (CIT) program supports a change in the research trajectory of junior faculty, early career researchers, and doctoral students toward Community-Based Participatory Research (CBPR). CIT is within the Community Engagement and Health Equity Core (CEHE) at the Colorado Clinical and Translational Sciences Institute (CCTSI), an NIH-funded Clinical and Translational Science award. This Translational Science Case Study reports on CIT’s impacts from 2010 to 2019. A team from The Evaluation Center at the University of Colorado Denver utilized four primary data sources: administrative records, participant written reflections, participant and Community Research Liaison (CRL) interviews, and community partner surveys. Data were analyzed using the framework of CBPR principles and the conceptual logic model. CIT trained 122 researchers in CBPR through embedded education within various Colorado communities. CIT Alumni secured ∼$8,723,000 in funding between CCTSI Pilot Grants and external funding. Also, CIT alumni implemented CBPR into curricula and community programming and developed deep, lasting relationships. Further key learnings include the crucial role of CRLs in building relationships between university and community partners and how CIT may serve as a mechanism to improve historical mistrust between communities and universities.

## Introduction

Established in 2008, the Colorado Clinical and Translational Sciences Institute (CCTSI) at the University of Colorado represents a pivotal effort in linking innovative scientific research with health advancements. Within this framework, the Community Engagement and Health Equity Core (CEHE) is critical to increasing the reach, effectiveness, adoption, implementation, and maintenance of clinical and translational research and aims to give communities a voice in the research that is important to them. The CEHE core focuses efforts on community engagement (CE) and Community-Based Participatory Research (CBPR), so researchers and partner community members gain foundational skills, apply relevant practices, and have ongoing and responsive support throughout the research and engagement process. Apart from the consistent emphasis within the Requests for Applications from the National Center for Advancing Translational Sciences for Clinical and Translational Science Award proposals for community engagement as a core activity, the challenges faced by faculty to pursue careers in community-engaged research is well documented [1,2].

Initiated in 2010 by the CEHE core, the Colorado Immersion Training in Community Engagement (CIT) program aims to introduce an expanded pool of researchers to CBPR, supporting a change in the research trajectory of junior faculty, early career researchers, and doctoral students toward community engagement [3]. CIT creates an infrastructure for educating researchers from a variety of disciplines. Training researchers in the foundations of CBPR aligns with the Clinical and Translational Science Awards initiative of the NIH Roadmap to accelerate the translation of discoveries into everyday practice [4] and is critical to ensuring that health research is not only scientifically rigorous but also culturally sensitive and relevant to community needs. While CIT provides an overview of the community engagement/community-engaged research/CBPR continuum, the focus on CBPR should equip researchers well for engagement along the continuum.

This paper reports the impacts of CIT in its first 10 years (2010–2019). It seeks to assess how effectively CIT has expanded the network of academicians involved in CBPR and understand its broader implications on future programming.

## Materials and methods

CIT uses a comprehensive approach of didactic learning and online discussion, experiential learning in partner communities, and Work-in-Progress meetings. The 6-month program is offered once a year at no charge and focuses on between two and five urban and/or rural communities (called community tracks), depending on yearly program funds. Program components include an orientation; 4 weeks of didactic curriculum and discussion about CBPR, historical and current events, and cultural aspects of partner communities; a week-long immersive experience in the partner communities led by CEHE Community Research Liaison (CRL) track leads and local community guides; and 3 months of Work-in-Progress meetings (see Table 1). Often CRL track leads begin mentoring relationships with participants during CIT which last well beyond the program.

**Table 1.** Colorado immersion training in community engagement activities outline – general 6-month timeline. This table presents the basic outline of a full 6-month colorado immersion training in community engagement (CIT) timeline, including components of the week-intensive agenda. CRL = Community Research Liaison; CBPR = Community-Based Participatory Research

Month	Activity/-ies	Description
Month 1	Orientation	Once accepted into the program, the orientation is an in-person session that brings participants together to meet each other, Community Research Liaison (CRL) track leads, and the CIT program staff. Participants are provided with detailed information about the program timeline, requirements, and expectations.
Month 2	Online curriculum and discussion	Participants are introduced to the fundamentals of community engagement and Community-Based Participatory Research (CBPR) during this 4-week didactic reading and discussion section. In addition, participants learn about their chosen community’s history and geography, its residents, culture, and socioeconomic profile through CRL chosen articles, novels, poems, and videos. Didactic components are accompanied by interactive online discussions.
Month 3	Week-intensive community immersion	The week intensive, also called the community immersion, begins with a half-day session with other participants, CIT program staff, and CRL track leads. Participants spend the next 5 days with CRL track leads out in community where they connect and interact with residents and community leaders, visit community-based organizations and businesses, and attend cultural events. The week intensive is largely spent away from academic settings. Participants must commit to being fully immersed in community for five full days and evenings of learnings, activities, and events. Depending on the community track participants select, this could mean staying overnight in the Colorado community. Example week intensive activities: Organized by CRL track leads with additional support provided by local community guides, each community track allows participants to: Explore first-hand the history, geography, and culture of a particular community; Connect with residents, groups, and organizations, for example, leaders and providers of physical, mental, behavioral, and public health services; spiritual leaders and healers; health and human services; pre-k-high school and higher education schools; public and tribal governments; farmers, ranchers, livestock producers; law enforcement; retail business, and others; and Gain skills in engaging communities in research.
Months 4–6	Reflection and work-in-progress	Participants come back together for three sessions, one per month, to reflect on their experience, receive mentoring from program staff, track leads, and community experts to facilitate continued communication and interaction between the participant and their host community, and learn about funding opportunities for CBPR projects. Track leads and program staff assist with identifying partnership opportunities and next steps. CIT participants may also begin mentoring relationships with track leads which often lasts well beyond the program.

CIT is advertised on the CEHE website, through campus newsletters, and by word of mouth and recommendations from CIT alumni, CRLs, and CEHE program staff. CIT participants apply for the program, identify their preferred track among those offered, and go through an interview process. Applicants must be housed at the University of Colorado Anschutz Medical Campus or another CCTSI-affiliated institution and be able to write grants. Candidates will, preferably, be continuing their work post-CIT in Colorado communities. Accepted participants are chosen by CEHE faculty and CRL track leads based on academic focus, expressed interest in CBPR, and track availability. A more detailed description of CIT was previously written by Zittleman *et al*. [3], and a version of the 2019 CIT curriculum is included as a Supplemental Material 1.

CRL track leads are a critical component of CIT, as multiple CRLs co-developed the program with CEHE staff. CEHE generally has between 8 and 10 CRLs on staff. The CRLs live in geographically dispersed regions of Colorado, represent diverse demographic and social characteristics reflected in Colorado, and were hired because of their extensive community leadership, advocacy, networking, and collaborative excellence with community partners. CRLs receive training in CBPR and translational research and build bridges between health research and community health initiatives while educating others about the purpose and value of equitable and participatory research partnerships. CRLs often assist research investigators in designing locally relevant research studies addressing community needs and facilitate bidirectional communication, partnership development, and culturally immersive learning experiences, including as CIT track leads CRL’s efforts are instrumental in fostering an environment of mutual respect and trust crucial for high-quality research endeavors and the establishment of lasting partnerships aimed at improving the health and well-being of underserved and underrepresented populations.

### Evaluation team and overview

The Evaluation Center (TEC) at the University of Colorado Denver conducted the evaluation of CIT. This evaluation, spanning a decade, was determined to be exempt from IRB review, and a 10-year executive summary is available [5]. Three primary methods were employed by TEC for data collection: document review, participant and CRL interviews, and community partner surveys.

### Document review

#### CIT participant reflections

Written reflections were requested from participants upon completing the program each year starting in 2014. Fifty-one reflections (out of a possible 55 participants during that period) were analyzed for evidence of prioritized program outcomes and suggestions for program improvement.

#### Administrative funding records

Funding records were reviewed to identify CCTSI grant funds acquired by CIT participants after participation in the program. Additional grant acquisition was viewed as one indicator of sustained research engagement.

#### CIT participant and CRL interviews

Starting in 2016, 42 semi-structured interviews were conducted with past participants 6 months to 2 years post-program completion to allow for the development of relationships and to actualize plans for CBPR outcomes. In 2020, four CRLs were interviewed for historical insights. All interviews were systematically recorded, transcribed, and thematically analyzed using NVivo software [6] focusing on specific program outcomes and emergent themes.

#### Community partner surveys

CRL track leads and program staff developed a survey to gather feedback from community members integrally involved with CIT to better understand the impact CIT had on their communities. The survey was administered in 2021 using Qualtrics^XM^ [7], an online survey platform. CRLs emailed invitations to their CIT community partners containing the survey link and the purpose of the survey. Respondents were offered a $20 gift card in appreciation for their time completing the five-question survey.

#### Analysis framework

Evaluation data were analyzed using the framework of CBPR principles and Wallerstein’s “Conceptual Logic Model of Community-Based Participatory Research: Processes to Outcomes” logic model [8]. This approach aimed to uncover shifts in researchers’ thinking related to how to conduct research and engage with community and actions taken toward implementing CBPR. The CBPR logic model facilitates a more comprehensive understanding of the program’s impact by outlining potential areas of impact in system change, capacity change, and improved health for communities. The logic model serves as a roadmap for considering how local context and broader social and cultural factors influence social outcomes.

Evaluators utilized the CBPR principles and domains of CBPR during interviews to probe for how researchers talked about the contexts and dynamics of attempting CBPR, as well as to probe for plans or actions taken to implement these learnings into research interventions. The elements of the logic model were also used in the thematic coding of participants’ reflections and the analysis of interview transcripts. These approaches were used to gauge shifts in researchers’ perspectives and actions and to assess long-term impacts, such as changes in policies, practices, power relations, sustained interventions, and improved health disparities and social justice.

Evaluators applied Social Cognitive Career Theory, which correlates increases in knowledge, confidence, and self-efficacy combined with meaningful experiences with stronger intentions and abilities to pursue a career path (i.e., CBPR-informed researcher). Evaluators applied this theory during semi-structured interviews to probe for researchers’ self-efficacy in conducting CBPR, their outcome expectations related to their research impact and career aspirations with a CBPR focus, and beliefs related to structural factors or barriers that may impact a career in CBPR. These theory components were also used during inductive and deductive coding of interviews to gauge researchers’ motivation to pursue a career in CBPR after their CIT experience.

## Results

### Program participation

In the first 10 years of programming, CIT engaged 122 academic researchers in CBPR training within 8 diverse Colorado communities. Most participants were from the University of Colorado; however, a few were housed at CCTSI-affiliated institutions such as University of Denver, Colorado State University, Denver Health, and Colorado Department of Public Health and Environment. Participant degrees and roles included MD, RN, PhD, NP, MSW, Assistant and Associate Professor, Research Manager, and Postdoctoral Fellow. Many disciplines were represented including Pediatrics, Behavioral Health, Epidemiology, Family and Internal Medicine, Neurology, Health Informatics, and others. Participant demographics, including education, title/role, etc., were not collected over the 10 years in a standardized consistent manner; thus, we do not have complete data. Table 2 shows the number of participants by focus community track offered each year. Throughout CIT, researchers were able to renew or establish perspectives and practices toward community-centered methodologies [9–12].

**Table 2.** Colorado Immersion Training in Community Engagement (CIT) tracks and number of participants by year. This table provides a yearly overview of the various community tracks and the number of academic participants involved. It effectively showcases the program’s reach and diversity over time, highlighting its extensive engagement with different communities. LGBTQI+ = Lesbian, Gay, Bisexual, Transgender, Queer/Questioning, Intersex

CIT tracks by year and community									
Year	Rural Eastern Plains	Rural San Luis Valley	Urban African American	Urban American Indian	Urban Latino/a/x	Urban Asian Refugee	Rural American Indian	LGBTQI+	# Participants
2010	X	X	X	X	X				19
2011	X	X	X	X	X	X			24
2012	X	X	X	X	X	X			14
2013		X	X		X				12
2014		X		X		X			12
2015		X			X				9
2016					X	X			9
2017		X					X		8
2018							X	X	9
2019					X			X	8
TOTAL	3	7	4	4	7	4	2	2	124*

*Note* *Two academics participated twice; 122 individual participants.

#### Research productivity

Analysis of research funding received by CIT alumni is an important indicator of the program’s impact. After CIT participation, 23 alumni partnered with community members and/or organizations to receive a CCTSI Community Engagement Pilot Grant. Eleven partnerships received a Partnership Development Grant, a small investment to support academic and community partners to further develop their relationship and explore the potential of a research collaboration. Five alumni with developed community organization partnerships received Joint Pilot Grants, a larger amount for established partnerships to produce preliminary data related to clinical or community interventions in preparation for non-CCTSI external grants. Seven additional researchers and their community partners received both Partnership Development and Joint Pilot funding. These 23 CIT alumni/community partnerships were awarded approximately $4,242,000 in follow-on non-CCTSI grant funding to support their community-based research. In addition, four CIT alumni (two of whom had not received Partnership or Joint Pilot awards) participated in other CCTSI Translational Pilot Grant programs and subsequently received another $4,481,000 in non-CCTSI funding.

In summary, 25 CIT alumni received 33 CCTSI Pilot Grants (both Community Engagement Pilot and Translational Pilot) and were subsequently awarded just over $8,723,000 in non-CCTSI funding.

#### Career aspirations

Participants reported that the program was instrumental in nurturing new knowledge, providing key contacts and support, and reinforcing the participants’ interest in pursuing CBPR-related work, as predicted by social cognitive theory [9]. For instance, one participant noted that CIT facilitated discussions about the mentor–mentee relationship and identified specific aspects to be aware of when transitioning from a mentee to a mentor role. Another highlighted how the immersion week helped in identifying public health research interests and potential community partners. The impact of CIT on fostering an appreciation for qualitative methods in the research process, particularly storytelling traditions, was also evident in the feedback. Additional examples and quotes are found in Table 3.

**Table 3.** Colorado Immersion Training in Community Engagement program outcomes – participant perspectives. Findings, themes, and quotes. This table presents key themes and quotes from qualitative analysis of participant interviews and written reflections, including the program’s impact on participants, suggested program enhancements, and challenges experienced in pursuing Community-Based Participatory Research (CBPR). CIT = Colorado Immersion Training in Community Engagement; CBPR = Community-Based Participatory Research

Finding or theme	Example story or quote
**Impact of CIT on participants**	
Career aspirations – knowledge gain, relationship building, and reinforced interest in CBPR-related work	“I found the program opened the door for discussions about each aspect of the mentor-mentee relationship, and, importantly, has shown me specific things to be aware of as I transition from mentee into mentor.” -Anonymous CIT Participant “The CIT program on the whole introduced me to CBPR principles and concepts, while the immersion week helped me identify my public health research interest and people that I can potentially work with in the community…The CIT experience definitely spurred my interest to work further with the refugee population…” -Anonymous CIT participant
Career aspirations – qualitative methodology and storytelling traditions	“I’ve never been a real qualitative methods person. I think that part of the research methods world is really now something I’m very interested in and really hope to pursue in a much greater and more significant way.” -Anonymous CIT participant
Cultural humility	A former CIT participant highlighted the importance of respecting community perspectives: “It’s just that knowledge of being really, really careful not to come in with my own values…What do you all need? Is there anything that I can help?” and another added, “You realize that your perspective isn’t enough -- that you need other people’s perspective… that we all are shaded by our own perceptions. The more people that we can engage, the better our research is going to be.”“A lot of good conversation happened during these times [unstructured, sharing of meals], more so than during the formal discussions.” -Anonymous CIT participant“I gained insight that there’s no way I could have gained just from reading a book. I gained exposure to people, circumstances, and stories that were moving and engaging. Having the opportunity to get out of our everyday environment and really listen and learn from others who are so passionate about their involvement was a huge encouragement and wonderful opportunity that I’m very grateful to have been a part of.” -Anonymous CIT participant
Commitment to the community	“I now understand that doing community-engaged research is doing research from the heart. To do this type of research, one needs to truly believe in what this can accomplish and to be authentically committed to it for what it is and not for the professional benefits it might bring.” -Anonymous CIT participant“Another beautiful story is that even some of our graduates that went through CIT, we told them, “Even if you do not go for a grant, if you’re not ready right now, there are other things in community where you can use your expertise.” Some of them got on boards of directors… That commitment from past participants and other partners helping these community-based organizations was a win-win. It’s great for them to be seen in community, and it’s great for the organization to have their expertise and have that comfort level built on one another…it really adds to the perception that change in a lot of the participants, a comfort level is established during that week where they’re in and out of community.” -Anonymous Community Research Liaison
**Suggested program enhancements**	
Structured opportunities to stay connected with current and past participants	“I would love to have more opportunity with previous cohorts to meet and to really gain insight from their experience. I feel like I need that now. I just feel like I personally could really benefit from a structured opportunity to continue to connect once we’ve wrapped it up. I think it would help keep my momentum.” -Anonymous CIT participant
Mentoring post-CIT	“I would love more support about how to tailor grants for organizations that don’t understand CBPR. A workshop … “Let’s invite all our past graduates to come together and talk about this topic in particular,’ would be awesome.” -Anonymous CIT participant
Maintaining connection with communities	“I think one of the biggest supports that needs to be in place is … having more opportunities for [researchers] to connect with members of the community in both formal and informal settings to build rapport and trust.” -Anonymous CIT participant
**Challenges pursuing CBPR**	
Protected time and institutional value	“The challenge that I’ve run into is a lack of understanding of what community-based research looks like among colleagues, [I get] comments or questions about, “You don’t seem to be around very much.’ That’s because I’m out in the community…We’ve spent a lot of time at our partner organizations, rather than in my office. At the university … it’s a challenge in the sense of feeling like my work is valued, and that it’s okay to not be at my desk on a day-to-day basis. That’s a really hard challenge to address. You cannot give everybody CIT training to make sure that everybody understands the work that we’re doing.” -Anonymous CIT participant“You have to put in your time initially and then work towards getting bigger grants, but none of my time is actually covered for any of this work, so it’s a balance as a clinician researcher trying to balance all my clinical and teaching responsibilities along with doing these projects at the same time.” -Anonymous CIT participant“Just lack of protected time to do it. Unless it’s on your “grid” you have to fit it in in between the seams. I could do that a lot more before I had a kid. This work takes time, and in order to make sure that this work gets done you have to cover that time somehow, and right now there really isn’t any funding for that. We’ve had to create it ourselves through leftovers from different projects. That’s the tact that we’ve taken.” -Anonymous CIT participant
Funding streams for CBPR	“I think the challenges there are mostly on the funding agency side. My experience is…they want to have community and patient stakeholders at heart, but they got so caught up on their comparative effectiveness that sometimes they’ll throw the baby out with the bathwater.” -Anonymous CIT participant“Folks have then gone back to their respective places, had conversations, and changed internal policies about what research looks like. I’m hoping that people being on these teams and having these experiences have these softer outcomes of better patient treatment, or other partnerships, or policy. There’s a ripple effect that this is bigger than just a research project…Those aren’t measurable at this point. There are lots of outcomes that you can’t measure—stories of what equity looks like or what relationships look like—these are bigger, and that is a test of how community wins.” -Anonymous CIT participant

#### Deepened cultural humility *and* commitment to community

A profound outcome of CIT is the shift in participants’ perspectives, particularly in terms of cultural humility, [13–15] and a deepened commitment to community engagement. Participants widely reported a deepening of their understanding and appreciation for cultural humility in conducting research with communities. This change was evident even in researchers who previously had experience with CBPR or community-engaged initiatives.

CIT alumni came to appreciate that CBPR is fundamentally higher quality, due to the diversity of viewpoints and the shift in power dynamics it entails, than traditional research methods. The more informal aspects of CIT, such as sharing meals and participating in daily community activities, were highlighted as equally important as the structured components, providing opportunities for building collaborative partnerships and gaining insights into community life.

#### Participant suggestions for improving CIT

CIT participants suggested improvements to CIT that would enhance what they gained from the program. As noted in Table 3, evaluation findings consistently show that participants wanted structured opportunities to maintain connection with their cohort of participants and with past participants. They also suggested more educational offerings, mentoring, and more networking opportunities after the immersion experience. Many participants were eager to maintain a network of CBPR-focused individuals, through ongoing online forums, networking events, workshops, or additional mentoring on implementing CBPR.

#### Challenges in continued pursuit of CBPR

CIT alumni often faced significant challenges, particularly in aligning the intensive nature of CBPR with traditional academic structures [16–18], as indicated in Table 3. The most common challenge cited was navigating academic research, clinical, and teaching productivity requirements alongside the time commitment required for quality, community-driven research. Many participants noted their academic time did not typically allow for the intense time commitment required to develop impactful community-focused research. Some researchers felt their colleagues or departments did not fully understand CBPR or value bidirectional research relationships. Moreover, many outcomes of CBPR, such as tangible community benefits, were not adequately valued in academic circles, particularly in promotion and tenure considerations.

A related challenge commonly encountered by CIT participants was finding appropriate funding streams that align with community-based research. Researchers must frequently contend with grant requirements and funding streams that may not truly value the outcomes of CBPR or that insist on measuring CBPR against standard grant productivity measures ill-fitted to describe community outcomes. Community-based research may identify outcomes such as changes in community policies, repaired relationships, and community-oriented dissemination over academic outcomes, such as journal publications that may not be accessible to community partners. Lastly, evaluation findings consistently show that participants wanted more educational offerings, more mentoring, or more networking opportunities after the immersion experience, as noted in Table 3.

### CRL track lead perspectives

Interviews with four of the CRLs who designed and led tracks during 2010–2019 highlighted their role and perspectives of CIT. CRL track leads heavily influenced which tracks were offered each year. They worked meticulously with program staff and their respective communities to develop and execute track-specific activities and curriculum, including curating a selection of historical and current track-specific readings and materials for the didactic component; meeting with community residents and organization leaders to discuss participation and plan visits; and arranging access to places and events of importance to communities. This was possible because of CRLs’ years-long efforts to develop and nurture deep relationships with individuals, organizations, and community groups in their respective communities. CRLs guided researchers’ learnings through exposure to stories, ways of knowing, cultural practices, and beliefs not often accessible to biomedical researchers, thus supporting a shift in traditional academic mindsets toward the values and worldviews present in the community. As noted by the quotes in Table 4, CRLs had expectations of how researchers show up in their communities and witnessed researchers struggling with aligning textbook instruction with often very different realities experienced while immersed in communities.

**Table 4.** Colorado Immersion Training in Community Engagement program reflections – Community Research Liaison track leads and community partners. This table presents key themes and quotes from qualitative analysis of Community Research Liaison (CRL) interviews and responses to open-ended survey questions asked of community partners engaged in Colorado Immersion Training in Community Engagement (CIT) over the 10-year period. LGBTQ = Lesbian, Gay, Bisexual, Transgender, and Queer

Finding or theme	Example story or quote
**CRL track lead reflections**	
CRLs develop and execute CIT track-specific activities and curriculum – often like a facilitator, sometimes even facilitating researchers’ painful unlearning of problematic ways of conducting research.	“This is not a show for people to just come and learn about the communities. These have to be serious researchers. They have to prove that really do want to work with community, and they’re not just “drive by.” I don’t use that term loosely. We’re not on display… Sometimes it’s beautiful. My role is [to] move them forward and facilitate their learning. It’s not to judge them. It is hard though to see some of that. My role is also to make sure that they don’t do harm to communities, so making sure that, if something is said or done, we address it immediately.” –Anonymous CRL
CRLs foster an environment of mutual respect and trust – with the goal of promoting high-quality research endeavors and lasting partnerships.	“There was a lot of mistrust towards the University in the beginning, but it was the liaisons who helped earn that trust back. The community that we’ve been working with over the years, gets it. That’s why they’ve been able to and feel comfortable applying for grants or being supportive of the work that we’re doing. … That’s taken some years to develop and to earn that trust, but it’s there now.” -Anonymous CRL
CRLs understand nuanced historical and generational differences in certain populations.	“I see people having cultural conflict honestly with the training, with their education that they’ve had. There are training components that I see them struggle [with], and that maybe they have had some education around maybe certain populations… or no experience with working with a community. I watch them be vulnerable. I watch them be challenged. I’ve heard comments that have been enlightening. I’ve witnessed people crying and checking themselves.” -Anonymous CRL“This is an eye opener… Of course, the researchers have to put themselves in the refugee’s shoes. They do not come with suit and tie and say, “Hey, I’m the researcher. I’m going to analyze your situation.” No, no, no.” -Anonymous CRL
**Community partner outcomes**	
Components needed for continued community–academic relationships	“It was through this relationship that the students, families, and Chicano community became actively engaged with CIT students to experience the actualization of our Freirean Liberatory education model. Understanding that people can be passive recipients of knowledge — whatever the content — or they can engage in a “problem-posing” approach in which they become active participants. As part of this approach, it is essential that people link knowledge to action so that they actively work to change their societies at a local level and beyond.”-Anonymous community partner“Trust level, confidentiality, intentions, and resourcefulness are all important factors to maintaining continued relationships.”-Anonymous community partner“Relationship building takes a lot of time - so a 1 week immersion is wonderful, but continued support for fostering these relationships would be helpful.” - Anonymous community partner“Provide more communication to stay engaged and stay up on what’s new with the CIT and partner organizations.” - Anonymous community partner
Using history, location, and story to understand the past and current healthcare environment	“I worked with CRL to create a tour of LGBTQ history in Denver for their cultural competency. The tour, which focused on the history of LGBTQ healthcare, intersected with stories of non-profits in the Capitol Hill neighborhood of Denver. … Our tour looked at the history of the Colorado AIDS Project (CAP) from 1984 - 1987, and yet how CAP lived in the very neighborhood where people were doing survival sex work, how they faced police violence and societal rejection, and how the sexually transmitted infection clinic at Denver Health was one of the few places they could get free/equal access to healthcare. I pulled examples of case studies from how health providers went into gay bath houses and did STI testing, and in Denver’s case how the local group Colorado ACT UP, helped to get attention and funding for HIV/AIDS from 1987 – 1990. … They learned how the space and story were intimate to the healthcare.”-Anonymous community partner
Relationship building and collaboration through experiential activities	“Not only can the CIT programs bring community leaders and researchers together, it can also help keep communities together moving towards more positive and healthy outcomes. That’s because programs like CIT increase collaboration, problem solving, and validate community concerns. The collaboration is that people bring their own knowledge and experience into the process. Training is typically undertaken in small groups with lively interaction and can embrace not only the written word but art, music and other forms of expression in realizing solutions to critical issues.” -Anonymous community partner“Good relationships with the Community Research Liaison and an exceptional opportunity to bring queer history to healthcare.” -Anonymous community partner

### Impact of CIT on communities

CIT would not be successful without the partnership of community organizations and individuals who live and work in the track communities. They are well known and respected in their community and engaged in CIT because of their close relationship with CRLs. These partners served in CIT as historians, story tellers, and frontline providers of resources and support for the communities they serve. Thirteen of these formal and informal community leaders from the Urban LGBTQIA+, Asian Refugee, and Latino/a/x, and Rural American Indian tracks were included in the evaluation on the impact of CIT on their communities.

Overall, 78% of community partners reported being “extremely satisfied” with their CIT experience. Just over one-quarter of partner organizations indicated they had not participated in research prior to CIT, with 36% not having participated in CBPR.

Forty-five percent of community partners were aware of research occurring that was a result of relationships built between CIT participants and community members, and 64% were aware of collaborations or projects that had developed because of CIT. Additionally, 55% of community partners were aware of ongoing communication between CIT alumni and their community or organization. Over one-quarter of community partners reported that healthcare or clinical practices in their community have changed “a moderate amount” or “a lot” to be more accessible since partnering with the CIT. Additionally, 64% of surveyed partners reported community members or organizations within their community having a more favorable opinion of university research because of CIT.

Table 4 includes experiences with CIT noted by community partner respondents to open-ended survey questions, in which community partners identified components needed for continued community–academic partnerships and suggested improvements for CIT. These included more opportunity post-CIT for relationship building and continued communication about CIT from CEHE. While change in trust was not quantitatively measured in community partner surveys, the theme of trust routinely surfaced in interviews with CIT participants as well as with the CRLs. As noted in Table 4, one CRL described trust issues with the University of Colorado and work CRLs did to improve trust among themselves and the university followed by trust among community partners and the university. In addition, as noted above under research productivity, many of the community partners engaged with CIT alumni on CCTSI Pilot Grants and funded projects, and some community partners have served on the CEHE governance council, all of which require trusting relationships.

## DISCUSSION

CIT’s success in creating an effective educational infrastructure for academic researchers interested in CBPR is evident from the results described. Over its decade-long implementation and extensive engagement in various communities across Colorado, CIT established its initial goals of creating a robust infrastructure for CBPR, expanding the pool of researchers skilled in CBPR from various academic and healthcare institutions and disciplines, and fostering long-lasting partnerships between researchers and communities.

An impressive 20% of CIT participants subsequently developed or enhanced existing community–academic research partnerships through CCTSI Pilot Grants, thereby increasing the research capacity of community organizations and engagement capacity of research institutions.

CRL track leads have the critical role of bringing participants into communities to learn from community leaders who may otherwise be inaccessible to participants. CRLs facilitate these community–academic relationships to build the research capacity of their communities and challenge researchers to embrace cultural ways of knowing and healing.

These impacts show that CIT is a valuable instrument for orienting researchers from traditional research backgrounds toward the benefits of CBPR through facilitation of bidirectional contributions, learning, and unlearning; challenging and transforming researchers’ perspectives; and empowering communities to take a leading role in research that affects them.

In addition to fulfilling many of the early predictions [3], CIT revealed unexpected outcomes. These were the incorporation of CBPR principles into academic curricula, impacting faculty career aspirations and perspectives, together with deep personal transformations. Several CIT participants have become involved in CEHE core programs, including serving as Pilot Grant reviewers, coaches for grant recipients, and members of the community–academic council overseeing the CEHE core. Many CIT participants have engaged with partners outside of their academic role, such as facilitating groups and serving on boards. Community partners serve as advisors to CEHE programming and present about their work at community–academic research forums.

These outcomes highlight the profound and multifaceted impact of the CIT program, extending beyond its immediate goals and reaching into the broader academic and community spheres.

### Addressing institutional and funding challenges

While CIT has successfully and positively impacted many researchers toward CBPR and facilitated funding of numerous community–academic partnerships, these results also highlight ongoing challenges CIT participants face within academic infrastructures. The academic research, clinical, and teaching demands of academic institutions and related performance metrics are not conducive to and do not measure time in communities building relationships, incorporating community ways of knowing, and focusing on community-prioritized research questions. As was mentioned in the Introduction, CIT focuses on the components of CBPR, which is further along the community-engaged research continuum than many participants may reach, due in part to time and funding restrictions. However, the training received in CBPR will serve them well in most any community–academic relationship.

This misalignment suggests a need for academia to broaden its evaluation criteria to include CBPR impact, especially for early career researchers who face significant pressure to meet existing academic metrics.

In addition, traditional funding structures often fail to recognize or value the unique infrastructure requirements and outcomes of community-based research, such as policy changes, relationship building, and community-oriented dissemination, which are often not covered under grant funding or under academic time [16–20]. This disconnect highlights the need for more flexible and inclusive funding models that can accommodate the distinct nature of CBPR and its focus on community-oriented outcomes [20,21]. Finally, as one moves along the spectrum of community-engaged research, the most effective community outcomes are those in which most of the investment goes into the community itself, rather than the university [19,20,22]. This misalignment underscores the need for structural changes in academia to better support and value CBPR. The challenges of protected time, especially for early career researchers balancing clinical duties and teaching requirements, further emphasize the need for institutional support for CBPR endeavors.

## Limitations

The evaluation of CIT largely relied on self-reported measures and retrospective data collection. While these methods provide valuable insights into the participants’ experiences and perceptions and are common for this type of evaluation, they are inherently subjective and may be influenced by recall bias. Additionally, the lack of a control group or comparative data limits the ability to make definitive causal inferences regarding the program’s impact on research productivity and community engagement. Administrative data collection tools and variables changed during the first few years of the program, thus limiting the ability to provide a complete profile of participants and community partners. These metrics have become more standardized over time.

The findings and outcomes may not be directly transferable to other regions or institutions. The specific cultural, socioeconomic, and institutional dynamics of Colorado may have influenced both the implementation of the program and the results observed, thus limiting the generalizability of the findings.

The evaluation covers a 10-year period, which, while substantial, may not fully capture the long-term impacts of CIT on alumni’s careers, community outcomes, and the sustainability of partnerships.

The focus on qualitative outcomes, though rich in detail and depth, is complemented by a less robust quantitative analysis. Metrics such as the number of grants awarded and external funding obtained, while impressive, do not fully encapsulate the broader impacts of the program, including changes in community health outcomes, long-term research collaborations, and institutional changes in support of CBPR.

## Future Directions

The future of CIT and similar programs lies in their ability to adapt to the evolving landscape of academic research and community voice in research. Indeed, our evaluations and feedback are an annual part of our process and have resulted in numerous modifications that include increasing the time for reflection, taking more care to ensure that interactions in community are not extractive and expanding participation to those in study coordination roles. Our curriculum is a living document, with adjustments made based on feedback and learnings from prior years. There is a growing recognition of the value of community perspectives and the need for more equitable power dynamics in research. Programs like CIT, which emphasize bidirectional learning and community ownership, are at the forefront of this change. CRLs, CEHE staff, and long-time community partners are building a “Research Readiness” curriculum for community organizations that want to increase their capacity for engaging in CBPR with academics. Given the challenges faced within academic settings, future directions should also include strategies to integrate CBPR more fully into institutional frameworks. This might involve advocating for policy changes, developing new funding structures supportive of CBPR, and promoting institutional recognition of the value of community-engaged research. CEHE has developed a free community engagement consultation service open to both academics and community partners and is working on bringing components of CIT into a variety of course curricula, including that of the Colorado School of Public Health.

## Concluding remarks

In conclusion, CIT represents a significant step toward integrating CBPR more deeply into academic research. Its success in training researchers, fostering community partnerships, and co-building programming with communities sets a precedent for other institutions. To replicate CIT’s success, institutions must embrace CBPR values, provide protected time, and establish funding mechanisms that support the unique demands of CBPR. This approach not only benefits academic research but also contributes to building healthier, more equitable communities.

## Supporting information

Wright et al. supplementary material Wright et al. supplementary material

## Supplementary material

The supplementary material for this article can be found at https://doi.org/10.1017/cts.2024.658.
