# Supplementary material for: Colorado Immersion Training in Community Engagement: Ten years of learning and doing
Source: J Clin Transl Sci. 2024 Dec 5;8(1):e227. doi: 10.1017/cts.2024.658 (PMC11713420; doi:10.1017/cts.2024.658)
Supplement: Wright et al. supplementary material [file S2059866124006587sup001.docx]

**Colorado Immersion Training in Community Engagement**

**CCTSI Community Engagement and Health Equity Core (v. 2019)**

**CIT Description:**

The Colorado Immersion Training (CIT) is a unique community-campus educational initiative that aims to introduce an expanded pool of researchers to community-based participatory research (CBPR) and community engagement. CIT supports a change in the research trajectory of junior faculty, early career researchers and doctoral students towards community engagement. CIT is a six-month program for researchers from all disciplines seeking new skills and knowledge in community engaged translational research.

**Course Objectives:**

- Learn core principles of community-based participatory research and community engaged translational research
- Develop skills to engage community partners in research
- Spend time in a local Colorado community to learn first-hand about the community and begin relationship-building
- Receive ongoing technical assistance and coaching from CCTSI Faculty and Staff
- Learn about special funding opportunities

**Program Components**:

The CIT Program has four major components:  1) An in-person orientation and introduction of participants, CRL track leads and administrative details; 2) online curriculum/discussion, consisting of readings, videos and assessments, paired with online group discussions; 3) a week-long intensive community immersion and 4) Work-in-Progress meetings on topics related to CBPR and funding.  These activities include writing a 3-page summary of the experience with CIT and completing an anonymous feedback form after completing the Week Intensive and attending a reflection meeting.

Week Intensive Community Immersion

Work-in-Progress Meetings

In-person Orientation

Online Curriculum and Discussion

Mentorship (as requested) provided by CRL track leads

Topics jointly set by CIT participants, CEHE staff and CRL track leads

Agenda set by CIT program staff and CRL track leads

Curated by CEHE staff and CRL track leads

Designed by CRL track leads; executed by track leads and community partners

**Helpful Resources:**You can read more about the CCTSI Community Engagement and Health Equity (CEHE) core here: https://cctsi.cuanschutz.edu/community

**Schedule of Online Curriculum and Discussion Questions**

**Note: each week during the online curriculum phase, track specific readings/materials will be provided in addition to the Core Readings along with questions to consider. These materials are hand selected each year by CRL Track Leads to educate participants on the history, culture, and current community strengths, opportunities and concerns of track communities. They include historical and current community documents, news videos, book chapters, community partner organization websites for review, and others.**

| **Colorado Immersion Training in Community Engagement (CIT)** | | | |
| --- | --- | --- | --- |
| Date | Topic | Discussion Questions | Readings |
| Week 1:  Core Readings | **What is CBPR and what is its role in health research?** | Please share your thoughts (**brief** is good--one paragraph is fine) on **one** of these questions relating to this week's readings:   1. What is your comfort level with respect to sharing control with community on important decisions related to the research process? 2. What excites you and what worries you about doing CBPR? | 1. Israel B, Schulz A, Parker E and Becker A. (1998). Review of community-based research: Assessing partnership approaches to improve public health. Annual Review of Public Health. 1998, 19:173-202. 2. Wallerstein N & Duran B (2010). Community-Based Participatory Research Contributions to Intervention Research: The Intersection of Science and Practice to Improve Health Equity. American Journal of Public Health, *100*, No.S1. |
| Week 2:  Core Readings | **Community Voice** | Dr. Syme talks about how “brilliant” we researchers ***think*** we are; how hard it is for us to view the community as an empowered partner, and how our approach to fixing problems doesn’t always get at the root cause. The Pinto et al article talks about the importance of researchers building trust in the community.   1. share an experience you’ve had working with community partners that touches on something raised in the Syme article and/or share some thoughts you have on how you’d go about building trust in the community (something identified as being very important in the Pinto et al article). | 1. Syme L. The Community as an Empowered Partner. Paper presented to the Communities in Control Conference. Melbourne, Australia, April 2003. (replace this one with Bruno’s paper) 2. Pinto R, McKay M, Escobar C (2008). “You've Gotta Know the Community”: Minority Women Make Recommendations About Community-Focused Health Research, Women & Health, 47:1, 83-104, DOI: 10.1300/J013v47n01_05. |
| Week 3:  Core Readings | **Is CBPR For *You?* And if so, how do you get started?** | 1. CBPR Curriculum: When you think about your personality (introversion vs extroversion, preference for leading vs following, preference for being in control versus sharing control, or some other aspect of your personality that you think comes into play with this), to what extent do you think community-engaged research is a fit? What might be a natural fit and what might be a challenge? How could you overcome those? | 1. Determining if CBPR is Right for You. Campus-Community Partnerships Online Curriculum, Unit 1 Section 1.4 2. Research from the Periphery, Chapter 2: Framework & Approach: Action Research & Participatory Action Research 3. Participatory Action Research Table: Assumptions, Commitments, Methods/Practices, Questions worth Asking |
| Week 4:  Core Readings | **Race, Privilege and Bias** | What surprised you about the Implicit Bias Test?  Would anyone care to share a story of a time when you were the initiator of and/or recipient of racism, prejudice, or privilege?  OR  Discuss your thoughts of the *Dance of Race & Privilege* article. Can you think of another analogy that helps describe CBPR? | 1. **IAT Implicit bias tests**:  <https://implicit.harvard.edu/implicit/>   (Click to continue as a guest under “Project Implicit Social Attitudes”, click to proceed, then complete at least 3 tests of your choosing)   1. Chavez V, Duran B, Baker QE, Avila MM, & Wallerstein N. **The Dance of Race and Privilege in CBPR** – Chapter 5 in *Community Based Participatory Research for Health*. 2. White Fragility Video: <https://www.youtube.com/watch?v=DwIx3KQer54> |
